# How the Western Diet Thwarts the Epigenetic Efforts of Gut Microbes in Ulcerative Colitis and Its Association with Colorectal Cancer

**DOI:** 10.3390/biom14060633

**Published:** 2024-05-29

**Authors:** Avisek Majumder, Shabana Bano

**Affiliations:** Department of Medicine, University of California, San Francisco, CA 94158, USA

**Keywords:** hydrogen sulfide (H_2_S), short-chain fatty acids (SCFAs), ulcerative colitis (UC), inflammation, diet, microbiome, cancer, therapy, Western diet, epigenetics, IBS, colorectal cancer, gut health

## Abstract

Ulcerative colitis (UC) is an autoimmune disease in which the immune system attacks the colon, leading to ulcer development, loss of colon function, and bloody diarrhea. The human gut ecosystem consists of almost 2000 different species of bacteria, forming a bioreactor fueled by dietary micronutrients to produce bioreactive compounds, which are absorbed by our body and signal to distant organs. Studies have shown that the Western diet, with fewer short-chain fatty acids (SCFAs), can alter the gut microbiome composition and cause the host’s epigenetic reprogramming. Additionally, overproduction of H_2_S from the gut microbiome due to changes in diet patterns can further activate pro-inflammatory signaling pathways in UC. This review discusses how the Western diet affects the microbiome’s function and alters the host’s physiological homeostasis and susceptibility to UC. This article also covers the epidemiology, prognosis, pathophysiology, and current treatment strategies for UC, and how they are linked to colorectal cancer.

## 1. Introduction

Over the years, the dietary habits of Western countries have changed significantly, with an increased consumption of semi-processed and ultra-processed foods [1]. These processed foods contain fewer plant-derived dietary fibers and more easily digestible carbohydrates, fat, emulsifiers (used for food processing), and other additives [1]. These changes in dietary patterns can impact microbial communities and often lead to a condition called dysbiosis [2]. The microbial community in the gut partially ferments and breaks down dietary fibers, producing short-chain fatty acids (SCFAs) and other metabolites [3]. These metabolites can influence various metabolic processes in the host. Studies have shown that microbial metabolic compounds can induce epigenetic reprogramming in the host’s genome [4]. This occurs by changing the pool of substrates used for methylation and acetylation reactions and by generating other compounds that affect the activity of enzymes involved in epigenetic modification [4]. These epigenetic modifications can persist through cell divisions and can be passed down without changes in the gene sequence [5].

Ulcerative colitis (UC), mainly associated with chronic inflammation and ulceration of the colon, is a significant risk factor for the development of colorectal cancer [6]. Different studies have found that UC patients who consume a high-fiber, low-fat diet show reduced inflammation and microbial dysbiosis [7,8]. Although the intestinal microbiota is sensitive to different environmental cues (including diet, stress, medications, etc.) and is associated with chronic diseases like UC, and colorectal cancer, so far, no study has been performed to consider all of these factors together to understand the underlying cause of this disease. Hence in this review article, we describe how the Western diet affects the gut microbial community, leading to epigenetic modification of the host genome. Moreover, we discuss how different microbial metabolites produced from the gut can induce pro-inflammatory signaling during UC. Additionally, we discuss the mechanism of development of UC and how it is linked to colorectal cancer. 

## 2. Outline of Ulcerative Colitis

### 2.1. Overview

UC is an idiopathic and chronic disease that is also a subtype of inflammatory bowel disease (IBD) [9]. This disease was first described by Samuel Colitis in 1959 [10]. It is an unremitting inflammation of the colon’s mucosal lining [11]. Inflammation in UC starts in the rectum and extends to the proximal segments of the colon [11]. UC leads to small ulcers on the lining of the colon, which can lead to bleeding, diarrhea, pus, cramping/abdominal pain, nausea, and extreme fatigue [11]. Earlier, the exact etiology of UC was not known, but now it is well established that it arises due to dysbiosis of the microbiota and the external environment may play a vital role [11]. The gut microbiome maintains our gut health by regulating the epithelial barrier [12]. It plays a direct role in influencing immune function by provoking a pro-inflammatory environment [12]. SCFAs serve as energy substrates for specific groups of bacteria in our colon and regulate homeostasis in the microbiome [13]. Previous studies suggested that the presence of fewer SCFAs in the Western-type diet can affect the microbiome’s composition [13]. Other than diet, factors like excessive use of drugs, alcohol, smoking, and mental stress can cause dysbiosis by killing beneficial bacteria [14]. As a result, the immune system is activated and targets its own tissue, increasing intestinal permeability and resulting in bloody diarrhea [15,16]. 

**Figure 1:**
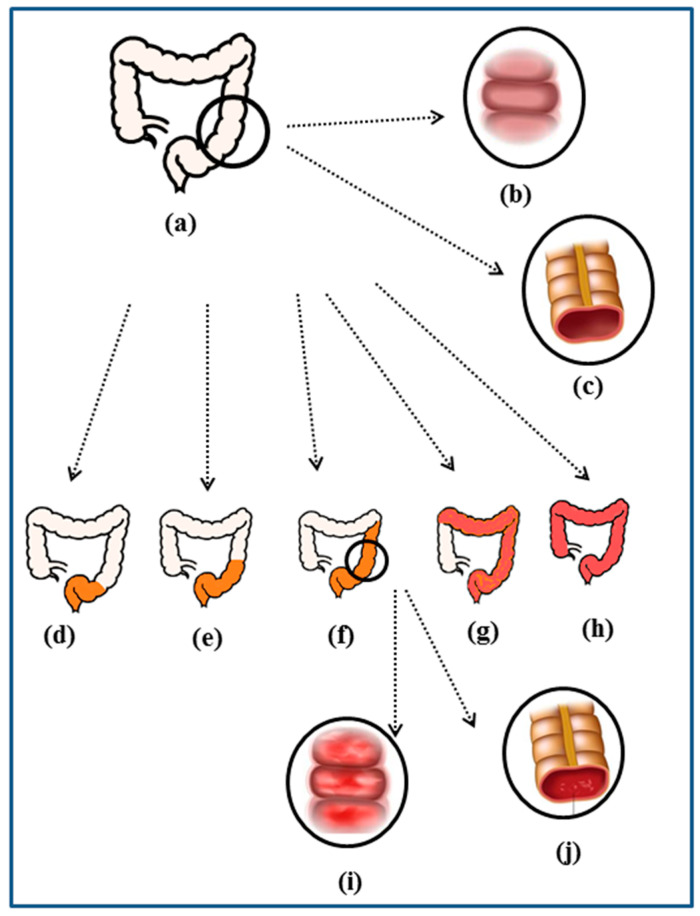
Anatomic distribution of UC. (**a**) Healthy colon, and (**b**,**c**) normal anatomy of the healthy colon. Different degrees of UC: (**d**) proctitis, (**e**) proctosigmoiditis, (**f**) distal UC, (**g**) extensive UC, (**h**) pancolitis, and (**i**,**j**) normal anatomy of UC.

UC is associated with significant morbidity in Western countries, and its incidence is increasing in developing countries [17]. This disease can develop in any individual group, but it is primarily diagnosed in the age group of 30–40 years [18]. This disease has two phases: one phase is called remission, in which diseased symptoms are under control, and the second phase is called the relapse phase, where disease symptoms are expressed, but this phase remains unpredictable [19]. Moreover, this disease is also associated with other manifestations affecting other body organs, like the eye, liver, skin, and joints [15]. Based on the location of the inflammation, UC is divided into five types: proctitis, proctosigmoiditis, distal ulcerative colitis, extensive colitis, and pancolitis (as shown in Figure 1) [20].

### 2.2. Epidemiology

The epidemiology of UC varies significantly worldwide. A report submitted by Molodecky et al., 2012 says that, from 1930 to 2010, the prevalence and incidence of UC increased worldwide [21]. The maximum incidences of UC have been reported in northern Europe (24.3 per 10^5^), followed by Canada (19.2 per 10^5^) and Australia (17.4 per 10^5^) [21,22,23]. The highest prevalence rates are recorded in Europe (505 per 10^5^), Canada (248 per 10^5^), and the USA (214 per 10^5^) [21,24,25,26]. Although recognition of UC is rising in Asia, the Middle East, and South America, very little data are available from these countries [27,28,29,30]. A study published by Sood et al., 2009 claimed that the prevalence rate of UC was 44.3 per 10^5^ inhabitants and the incidence rate was 6.02 cases per 10^5^ inhabitants in Punjab, North India, when the study was conducted [31]. So far, no sex predominance is reported in the occurrence of UC [32]. 

### 2.3. Prognosis

Recent data suggest that less than 10% of UC patients need colectomy within the first ten years of diagnosis [33]. Different factors like disease progression, certain levels of inflammatory markers, and age less than 50 years at diagnosis are associated with colectomy [33]. Modifiable risk factors associated with relapse of the disease are uncertain but could be induced by changes in diet patterns, mental stress, smoking, and poor adherence to drugs [34]. Although the disease may be associated with a modest increase in mortality in the community, this effect is attenuating in more contemporary cohorts of patients, perhaps due to earlier diagnosis and treatment improvement [35].

### 2.4. Risk Factors

The cause of ulcerative colitis is complex and involves many risk factors. Different clinical studies have found that age, race, ethnicity, genetics, and the gut microbiome are the main risk factors for UC [36]. However, other factors, such as lifestyle, diet, stress, overuse of certain drugs, exercise, and air pollution, can act as triggers for UC [37]. The risk of being diagnosed with UC is higher for individuals aged between 15 and 30, as well as for those older than 60 [38]. This suggests that age is one of the risk factors for UC. Some studies also proposed that people of white ethnicity, particularly those of Ashkenazi Jewish descent, are at greater risk of developing UC [39]. Additionally, having a first-degree relative with UC or Crohn’s disease increases the likelihood of developing UC, as up to 20% of people with UC have a family member who also has UC or Crohn’s disease [39]. These findings provide evidence that suggests that genetics play a vital role in the causation of UC. 

Emerging data from cohort studies in the United States and Europe have demonstrated an association between various environmental factors and the risk of UC [40]. Early-life events such as mode of birth (cesarean versus vaginal delivery), breastfeeding, and exposure to antibiotics, as well as factors such as air pollution, smoking, psychological state, exercise, and diet, are among the potential environmental contributors to IBD development or disease activity [41]. 

Studies found that antibiotics alter the gut microbiome composition, resulting in transiently decreased bacterial diversity, and cessation of antibiotics can return the microbiome to its pre-antibiotic state within ∼1 month [42]. Some studies have also shown associations between antibiotic use and IBD development but were limited due to recall bias [43]. 

Smoking plays a protective role against UC [44]. Smoking cessation dramatically changes the composition of and increases the variety of the intestinal microbiome [45,46,47]. Environmental pollutants like heavy metals could contribute to inflammatory diseases like IBD. Ingested mercury causes various disturbances in the intestinal tract, such as abdominal pain, IBD, ulcers, and bloody diarrhea [48]. Several studies have proved the association between significant life stressors, anxiety, depression, or psychiatric morbidity and the onset of IBD risk [49,50,51,52]. 

### 2.5. Current Treatment Strategies 

#### 2.5.1. Immunosuppressants, Steroids, and Biologics

Aminosalicylates are considered a treatment choice for mild to moderate UC, and topical and systemic steroids can be used to treat UC flares [53]. By contrast, immunosuppressants and biological drugs are used in moderate to severe disease [54]. In May 2014, the Food and Drug Administration approved vedolizumab, the first selective adhesion molecule inhibitor for use in moderate to severe UC when standard therapy has failed [55]. Vedolizumab is a humanized monoclonal antibody that inhibits the adhesion molecule a4b7-heterodimer, blocks leukocyte migration and the resultant gut inflammation, and is effective in inducing and maintaining remission in moderate to severe UC [11,56]. 

#### 2.5.2. Probiotics

Many probiotics have been used to treat various metabolic diseases [57,58,59,60]. Vsl-3 is one of these probiotics that has been evaluated in several studies [61]. It is efficacious in inducing remission in mild to moderate disease. However, data on its use as a maintenance therapy are poor [62,63], and data on the use of other probiotics are quite limited [31,64]. 

#### 2.5.3. Surgery

Surgery is introduced when *therapeutic* approaches fail (or corticosteroid dependence is indicated) or in the case of colonic dysplasia or cancer [65]. Approximately 10% to 15% of patients require surgical management of their disease [66,67]. 

#### 2.5.4. Fecal Microbiota Transplantation (FMT)

Fecal microbiota transplantation (FMT) is the most recent attention-grabbing treatment for UC [68]. This process involves restoring the microbiota by introducing a healthy mass of bacteria through the infusion of stool, e.g., via enema, orogastric tube, colonoscopy, or by mouth in the form of a capsule containing freeze-dried material obtained from a healthy donor [69]. Various studies have shown that FMT is a promising treatment with a high success rate [68]. 

## 3. Relationship between Environmental Inputs, Microorganism-Derived Metabolites, and Host Signaling Pathways in UC 

Microorganisms closely associate with eukaryotic organisms across the plant and animal kingdoms. Responding to environmental fluctuations, microorganisms quickly alter their transcriptomes, contributing to the host’s physiological homeostasis and disease susceptibility. Microbes and their hosts share the same environment, and microbial metabolic molecules (microbial metabolites) exert crucial effects on host physiology [70]. Environmental factors shape the composition of the host’s resident microbes and modulate their metabolism. In the following paragraphs, we discuss these relationships in detail. 

### 3.1. Gut Microbiome and UC 

#### 3.1.1. Composition of the Gut Microbiome

The number of bacterial cells found in the human gastrointestinal (GI) tract (GIT) is ten times the number of human cells and encodes 100 times more genes than the host’s genome [71]. This collection of all microbes, such as bacteria, fungi, viruses, and their genes, naturally living inside our body is known as a microbiome [72]. The colonization of the microbiota does not occur in the uterus, and the fetus, believed to be sterile or least microorganism-containing, develops microflora during the passage of the fetus through the vagina [71]. This is why the child’s gut shows a similar microbiota to the mother’s vaginal area when born by vaginal delivery. By contrast, babies born by cesarean section initially show fewer bacteria in comparison to infants who are delivered through the vaginal pathway [73]. However, these differences are not seen after 6 months of birth [73]. The bacteriology of the human GIT is extensive and beyond the scope of this review; we focus on how microbial metabolites can reprogram the host’s epigenetic code and how that relates to UC. In earlier times, studying the colon’s microbiota was extremely meticulous because culture-based methodology was used, and the culture of anaerobes was the hardest part. However, robust changes have occurred in microbiology techniques in the last decade by introducing culture-independent, high-throughput molecular techniques like 16S rRNA sequencing [74]. Data obtained from different studies show that around 2000 species can co-exist in the human GIT [75]. These microbes can be grouped into 12 phyla, of which approximately 94% belong to Actinobacteria, Bacteroidetes, Firmicutes, and Proteobacteria [75]. It was also noted that more than 380 identified species are anaerobic [76]. A study reported that a healthy person’s colon has about 500 different bacterial species belonging to 190 different genera [77]. Long-term colonization of microorganisms is suggested to be essential for normalizing the host’s colon mucous layer [78]. Although the mucous layer is present in the entire GIT, it is thickest in the colon, and this area plays a significant role in the relationship between the host and microbes [78]. The colonic mucous layer is divided into two different layers; the inner layer is impermeable and dense and is believed to be virtually sterile, while the outer layer is loosely structured and penetrable by microorganisms [75,79]. The outer layer contains mucin protein, which is decorated by the receptors of O-glycans, which provide binding sites and energy to commensal bacteria [80,81,82] (Table 1). 

#### 3.1.2. Role of the Gut Microbiome in the Causation of UC

A plethora of evidence has confirmed that dysbiosis of the GI microbiota leads to various diseases like IBD and other gastrointestinal disorders [83,84]. UC is a subtype of IBD, which also develops due to microbiota dysbiosis. Dysbiosis of the microbiota is a phenomenon where the alteration of the ecosystem of the microbiota takes place with respect to pathogenesis. A study by Machiels and colleagues (2014) compared the microbiota of UC patients (127 patients) over 85 years of age with sex-matched healthy people [85]. They found that the numbers of two bacterial strains, namely Raecalibacterium prausnitzii and Roseburia hominis, were significantly lower in UC compared to the control, and recently, another group confirmed this finding [85,86]. In another study, it was seen that alteration in the combination of the microbiota and disturbance in fermentation in IBS patients may play a crucial role in the development of symptoms of IBD patients, with a significant two-fold increase in the ratio of Firmicutes to Bacteroidetes reported in IBS patients [87]. In other studies, in UC patients, it was found that there was a decrease in bacterial diversity, a noticeable fall in the number of Firmicutes, and an increase in the number of Gammaproteobacteria and Enterobacteriaceae [88]. It has been seen in various studies that the gut microbiota maintains epithelial integrity and tight junction permeability. For example, *Lactobacillus plantarum* was reported to maintain the integrity of tight junction proteins against disruption of the epithelial barrier [89]. The loss of epithelial integrity permits gut bacteria, toxins, partially digested food, and waste material to pass through the epithelium into the bloodstream, which triggers the immune responses leading to gastrointestinal problems, such as cramps, excessive gas, abdominal bloating, and food sensitivities. These are the symptoms of a leaky gut, which displays hyperpermeability, resulting in bloody diarrhea [90]. 

### 3.2. Western Diet, the Microbiome, and UC

#### 3.2.1. Western Diet and Its Impact on Health and Disease

Before the agricultural revolution and animal farming, hominins’ main diet was limited to minimally processed wild plant and animal foods [91]. After the Industrial Revolution, different ultra-processed and refined foods were introduced that were not previously encountered in hominin evolution [91]. Hence, it is not hard to believe that adaptation to changes in diet may contribute to various chronic diseases [91]. In the US, approximately 65% of adults (aged 20 and above) are either overweight or obese, and obesity alone is attributed to a total of 280,184 deaths per year [92]. In an estimation, it was noted that about one-third of all cancer deaths are due to poor dietary habits and obesity [91]. These statistics undeniably illustrate the detrimental impact of the Western diet that diverges from our ancestors’ nutritional model. 

The Western diet is typically defined by the consumption of energy-dense but nutrient-poor processed and refined foods (like fast food and soft drinks), which are often high in added sugars, salt, and saturated fats [93]. By contrast, traditional non-Western diets, particularly the Mediterranean diet, contain whole grains, healthy fats, legumes, and a greater portion of vegetables and fruits [94]. Western foods are those foods that are altered from their natural condition to improve test, self-life, and energy value [93]. These foods also include processed meats (mostly red meats), which have been associated with risk for many metabolic diseases and colorectal cancer [95]. Furthermore, processed and refined foods comprise a significant portion of the typical Western diet, which mainly include cookies, cake, bakery goods, breakfast cereals, and snack bars [96]. It is important to note that dairy products, cereals, refined sugars, refined vegetable oils, and alcohol collectively account for 72.1% of the total daily energy consumed in the United States [97].

The Western diet and its adulteration with substances directly impact gut microbes and, consequently, human health [97]. During the agricultural process, some additives are used to increase productivity by enhancing resistance to pests and increasing crop yields [98]. In addition, during food processing, different additives (including preservatives, flavor, and sugar alternatives) are added knowingly or accidentally [99]. Common non-nutritive substances found in Western foods include pesticides, persistent organic pollutants (POPs), metals, and plasticizers [100]. These chemicals have been linked to different detrimental effects on our bodies. 

Animal-source foods are packed with vital vitamins and minerals crucial for good health [101]. Deficiencies in these nutrients can lead to serious health issues [102,103]. That is why the traditional rural American farm diet, mainly eggs, meat, and milk, has been associated with improved health and increased longevity.

#### 3.2.2. Western Diet Affects the Microbiome Composition in Relation to UC

Many factors shape the microbiota composition of the GI, but diet is one of the most important factors in maintaining the diversity of the microbiota of the GI [104]. It has also been noted that considerable changes in feeding habits increase the chances of UC [105]. Many epidemiological studies have proved this relationship between food and UC [90]. Two recent studies have shown that sucrose intake and soft drink consumption are related to the development of UC, and the risk of disease development was 10% and 69%, respectively [106,107]. Another study showed that consuming fruits and vegetables decreases the chance of UC development [106]. For example, children from rural areas of Africa show low counts of Firmicutes and Enterobacteriaceae and high counts of Bacteroidetes compared to children from European countries [108]. This variation is supposed to be due to the different dietary patterns of both populations [109]. Hence, it has been suggested that feeding habits can induce changes in the microbiota from a healthy state to a diseased state, which can further induce inflammatory diseases like UC [104]. A study also showed that an increase in pro-inflammatory bacteria and a decrease in protective bacteria could result from mucosal dysbiosis of the GIT, which is developed by consuming a high-fat/sugar diet [110].

**Figure 2:**
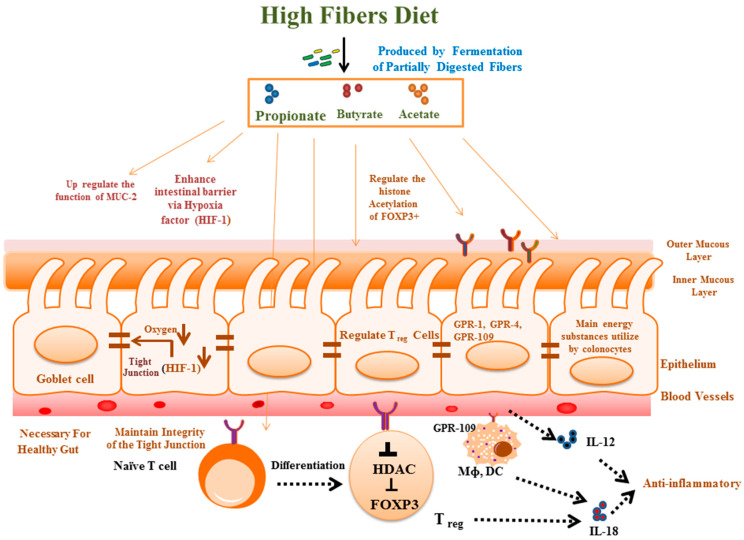
The role of food-derived SCFAs in the regulation of intestinal homeostasis: SCFAs serve as an energy substrate. In addition, they regulates various intestinal factors, like upregulating the formation of MUC-2 to maintain a healthy gut, maintaining the integrity of the tight junction, regulating Treg cells and various factors like GPR-1, GPR-4, GPR-100, and GPR-109, and activating the anti-inflammatory pathway.

Furthermore, diet pattern not only affects the microbial composition but also affects the metabolic function of microbes in the GI (Figure 2). Fatty acids with less than six carbons are called short-chain fatty acids (SCFAs), like formic acid, acetic acid, propionic acid, butyric acid, and valeric acid. SCFAs are derived from commensal bacteria when dietary fibers are partially digested [111]. More than 95% of SCFAs in the gut have less than four carbons, namely acetate, propionate, and butyrate [112]. These SCFAs play an essential role in immune modeling and maintain intestinal barrier integrity by controlling the mucosal barrier function of the GI [113]. Several cellular functions, like alteration of gene expression, cellular differentiation, apoptosis, and chemotaxis, and other functions of epithelial and/or immune cells are tightly regulated by SCFAs [114]. In some UC patients, it was found that levels of an essential SCFA-producing bacterium, *Faecalibacterium prausnitzii*, were decreased, and its activity was inversely proportional to the severity of the disease [85]. Moreover, in Western countries, people eat more sugar and less fiber, which decreases the survival of SCFA-producing bacteria and increases the chances of development of UC [110,115]. 

#### 3.2.3. Western Diet Thwarts Epigenetic Efforts of the Gut Microbiome in Relation to UC 

**Figure 3:**
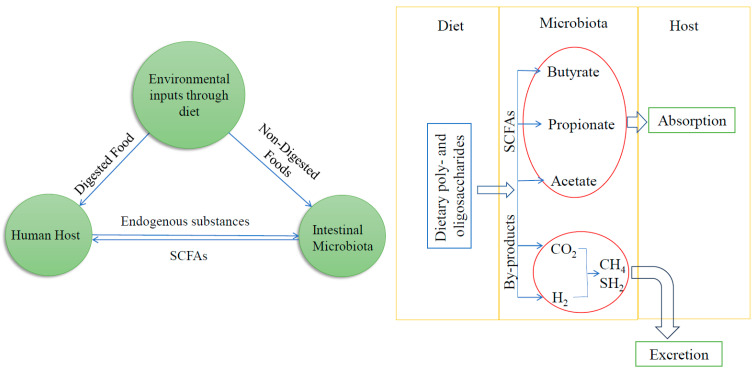
On the left side, this diagram depicts the intricate relationship between environmental inputs, microorganisms, and the human host. On the right side, it shows how the gut microbiome can use undigested dietary poly- and oligosaccharides and further process them to SCFAs and other by-products (CO_2_, CH_4_, SH_2_, etc.); then, SCFAs can be absorbed by the human host, and other by-products can be excreted from the host’s body.

The gut microbiota produces a variety of metabolites that can be detected in the host’s circulation, including small organic acids, bile acids, vitamins, choline metabolites, and lipids [116]. When the mammalian host cannot digest certain dietary poly- and oligosaccharides, they pass to the distal gut, where they become carbon and energy sources for gut bacteria [116]. Through fermentative reactions, the gut microbiota can metabolize complex carbohydrates to produce small organic acids, mainly comprising SCFAs like acetate, propionate, and butyrate (≥95%), as shown in Figure 3 [3]. Different preclinical studies have shown that microbes communicate with their host by sending out metabolites that act on DNA methylation, post-translational modification of histones, and regulation of non-coding RNAs, thus influencing gene expression in the colon and tissues in other parts of the body [117]. In the following paragraphs, we discuss these epigenetic modifications through the microbiota in detail. 

##### Regulation of DNA Methylation via the Gut Microbiome

DNA methylation is a process of adding methyl groups (-CH_3_) onto the cytosine residues in DNA, thus affecting gene expression. In DNA methylation, the methyl group comes from the methionine cycle [118,119]. During the methionine cycle, first the amino acid methionine (which comes from diet) is converted to S-adenosyl methionine (SAM), then different DNA methyltransferases (DNMTs) transfer the methyl group from SAM to cytosine residues of DNA, producing S-adenosyl homocysteine (SAH) [120,121]. In addition to methionine (an essential amino acid), metabolites such as folate, vitamin B12, betaine, and choline are essential for SAM production [122,123,124]. Different studies have found that microbial metabolism absorbs these metabolites [125,126]. A study reported that SCFAs (derived from the microbiota, as mentioned above) can regulate the phosphorylation of ERK and cause downregulation of DNMT1 and, consequently, demethylation of tumor suppressor genes, including RARB2, p21, and p16 [127]. 

##### Regulation of Post-Translational Modification of Histones via the Gut Microbiome

The eukaryotic genome is organized into a highly compressed nucleo-protein structure known as chromatin, where DNA wraps around histone proteins and folds to form higher-order structures [128]. Different types of post-translational modifications of histones are acetylation, methylation, phosphorylation, SUMOylation, poly-ADP ribosylation, biotinylation, ubiquitination, citrullination, and proline isomerization, and these modifications can influence gene expression [129,130]. Acetylation occurs in the Lys residue of histone tails. It minimizes the positive charge of histone, reducing the interaction between histone and DNA; as a consequence, it opens the regulatory regions of genes so different TFs can bind and promote transcription. Whereas methylation either activates or represses transcription [131]. Histone acetyltransferases (HATs) transfer the acetyl group from acetyl-coenzyme A (acetyl-CoA) to the lysine residue of histone protein, whereas histone deacetylases (HDACs) reverse this reaction [118]. Increased availability of acetyl-CoA can upregulate HAT activity, and studies have found that gut microbiota-derived metabolites SCFAs can modulate acetate (acetyl-CoA) levels [132]. Microbiota-derived SCFAs were also found to inhibit HDAC activity in the host tissue [132]. A study showed that HF/HS feeding of mice reduced SCFA production from the gut microbiota, leading to decreased global histone acetylation in the liver and adipose tissue of the mice [117]. This study also found that HF/HS feeding altered the methylation signature of histone in the mice. Many other studies have reported modulation of the post-translational modification of histones in colonic cells via microbiota-derived SCFAs, which led to differences in IBS [133]. 

##### Regulation of Non-Coding RNA via Gut Microbiome

Recently, different non-coding RNAs (RNAs that are not translated into proteins), such as microRNA (miRNA), short-interfering RNAs (siRNAs), piwi-interacting RNAs (piRNAs), and long noncoding-RNAs (lncRNAs), have been identified to be associated with various diseases [134,135,136]. A study identified the alteration of six lncRNAs in the intestinal epithelial tissues of mice when they compared germ-free mice with conventional mice (re-colonized with mice microbiota) [137]. In another report, when researchers compared the microbiota-mediated changes of lncRNAs in different tissues, such as in the liver, duodenum, jejunum, ileum, white adipose tissue, brown adipose tissue, colon, and skeletal muscle, they found significant alteration of lncRNAs only in the jejunum [138]. In an investigation, Virtue et al. identified the upregulation of miR-181a and miR-181b in epididymal white adipose tissues of conventional mice compared to germ-free mice [139]. 

#### 3.2.4. Western Diet Causes Colonic Inflammation through the H_2_S-Producing Gut Microbiome in Relation to UC

Excessive consumption of sugar and red meat (Western diet pattern) is known to increase the risk for UC, and a high-fiber diet and citrus fruits have been found to play a protective role in UC [140]. However, this association between the Western diet and UC is far more complex than we think. Other than distinct clinical features, inflammation of intestinal mucosa is the main characteristic of UC [140]. Studies have shown that excessive production of H_2_S from the microbiota in the context of reduced capacity of sulfide disposal in the mucosa may contribute to inflammation in UC [141]. The severity of the inflammation in the colon and rectum of UC patients is associated with a higher ratio of H_2_S-producing bacteria than other bacteria in the gut [142]. As the ratio of H_2_S-producing bacteria depends on the food available for producing these metabolites, the Western diet pattern may contribute to colonic inflammation by increasing the ratio of H_2_S-producing bacteria in the gut. A follow-up study of UC patients for one year showed that the relapse rate was higher in patients with high consumption of meat, dietary protein, and sulfur/sulfate than in patients with low consumption of these compounds [143]. This suggests that the intestinal bacteria may use these foods for H_2_S synthesis, which may associated with increased relapse in UC patients. Another study in a rat model showed that intracolonic instillation of NaHS (for 1 H at concentrations of 0.5–1.5 mM) increased the expression of inflammation-related genes like inducible nitric oxide synthase (iNOS) and interleukin-6 (IL-6) [144]. In a study, when the microbiota composition was analyzed in newly new-onset pediatric Crohn’s disease patients, they found enrichment of H_2_S-producing bacteria like Atopobium, Fusobacterium, Veillonella, Prevotella, Streptococcus, and Leptotrichia [145]. These genera of bacteria are known to produce H_2_S from sulfur-containing amino acids, and it has been proposed that excessive intake of sulfur (mainly from red meat) in the diet can lead to intestinal problems, including UC [146]. To investigate the direct association of H_2_S production via the gut microbiota and colonic inflammation, in an experiment, researchers colonized the H_2_S-producing bacterium *Atopobium parvulum* into the gut of IL-10 double knockout mice (a model of mice susceptible to colitis). They found worsening of colitis, whereas this effect was attenuated via treatment with an H_2_S scavenger, bismuth [145]. Additionally, the colonic mucosa biopsy results of the above-mentioned Crohn’s disease pediatric cohort showed lower expression of mitochondrial enzymes, which are involved in H_2_S detoxification [145]. 

**Figure 4:**
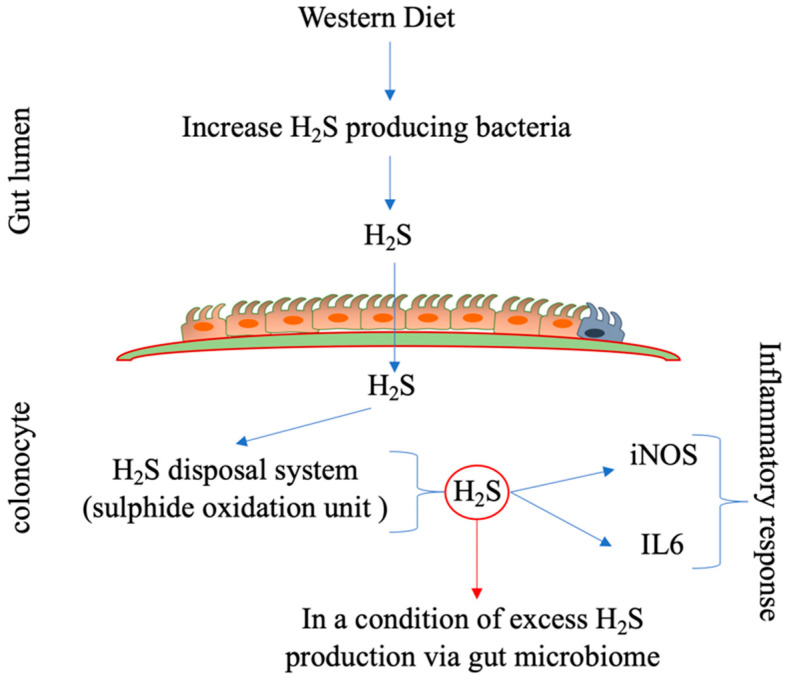
This figure schematically represents a condition of colonic inflammation via excessive H_2_S produced by the gut microbiota. The Western diet can increase the number of H_2_S-producing bacteria in the gut, and the excessive H_2_S produced by the microbiota can be absorbed by the colonocytes. If this absorbed H_2_S exceeds the H_2_S disposal capacity of the sulfide oxidation unit (SOU) of the colonocytes, it increases the expression of pro-inflammatory interleukin-6 and iNOS and causes an inflammatory response.

From the above preclinical and clinical data, it is evident that excessive production of H_2_S by gut microbiota (i.e., above the capacity of the intestinal mucosa to detoxify it) may cause colonic inflammation in UC, as shown in Figure 4. However, many recent studies have proposed that H_2_S is a beneficial gasotransmitter (it does not require a transporter protein) that can come from the intestinal microbiota and is produced endogenously via the trans-sulphuration pathway [147,148,149]. Different studies, either using H_2_S-releasing compounds or inhibiting H_2_S-producing enzymes, have proposed that minimal H_2_S is necessary to limit colonic mucosal inflammation risk [150,151,152]. H_2_S has been shown to have antioxidant capacity via scavenging of ROS and persulfidation of cysteine residues, and this activity of H_2_S may primarily contribute to its anti-inflammatory properties [153,154,155,156]. Endogenous synthesis of H_2_S was found to protect against dextran sodium sulfate (DSS)-induced colitis via inhibition of the inflammasome pathway [157]. Similarly, slow-releasing sulfide donor compound GYY4137 was reported to ameliorate lipopolysaccharide- or TNF-α/IFN-γ-induced increased permeability in the colonocyte monolayer [158]. Moreover, the releasing compound was found to improve mesenteric perfusion and intestinal injury in an experimental model of necrotizing enterocolitis via an eNOS-dependent signaling pathway [159].

## 4. Association of the Microbiome with the Modulation of Immunological Signaling in UC

**Figure 5:**
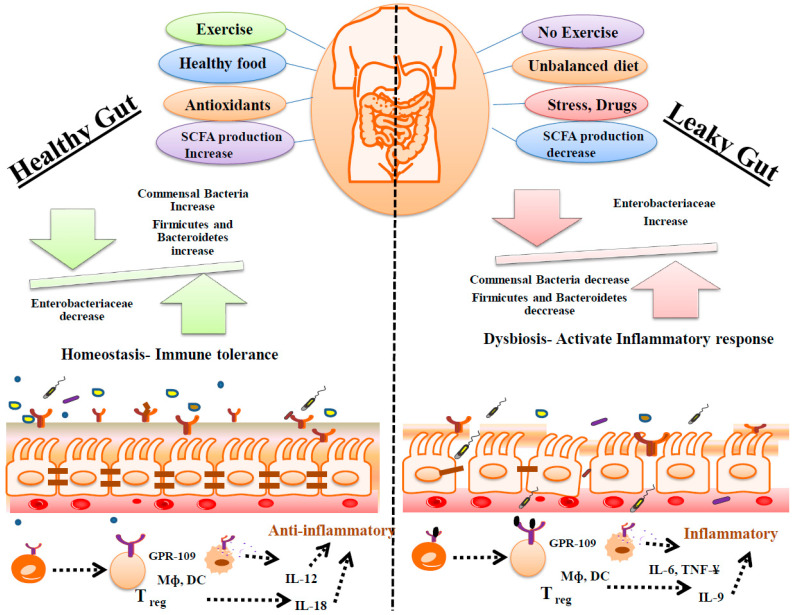
The interactions between various factors and the development of UC. Exercise, healthy food, antioxidants, and increase the production of SCFAs to maintain a healthy gut, but lack of exercise, unhealthy food, stress, excessive drugs, and decrease the production of SCFAs, which consequently leads to leaky gut or the development of UC. Triggering the immune response in UC, the main molecules involved are GPR-109, macrophage, dendritic cells, T regulatory cells, IL-6, IL-9, IL-18, and TNF-γ.

Both innate and adaptive immunity generate abnormal inflammation in UC patients [160]. The human GI tract has a mucous layer as the first physical barrier of innate immunity, which is composed of two layers, the inner and outer layers (Figure 5) [161,162]. The inner layer is considered sterile, while various commensal bacteria inhabit the outer layer. Next, the intestinal epithelium is considered the second barrier that checks the entry of bacteria, and it comprises enterocytes and specialized cells known as goblet and paneth cells [163]. Intestinal epithelial cells (IECs) protect the mucous barrier by blocking the influx of commensal bacteria and pathogens and secreting mucin and α defensin [164]. It also expresses antigen-sensitive receptors like toll-like receptors (TLRs) and nucleotide oligomerization domain receptors (NOD). Under healthy conditions, the intestinal epithelium is intact, but in UC, an impaired intestinal epithelium allows the entry of pathogens, and TLRs recognize the pathogens and activate the signaling of pro-inflammatory cytokines like interleukin (IL), IL-12, and IL-6 by IECs [165]. TLR signaling also regulates the activation of transcription factor NF-kB, which regulates the various genes responsible for innate responses like IL-1, -2, -6, and -12, and tumor necrosis factor-alpha (TNF-*α*) [166,167]. IL-1 and TNF-*α* are well known for their pro-inflammatory responses [168]. TNF-*α* plays a crucial role in the development of UC because it increases the expression of IL-1β, IL-6, and IL-33 [169,170]. It was also seen that the severity of UC was correlated with the level of TNF-*α* in the serum of the patients [171]. Besides the above-mentioned interleukins, other interleukins like 1L-8, IL-10, and IL-33 are also involved in UC pathogenesis [172]. A report showed that the IL-10 level in mucosal T cells was increased in UC patients compared to the control group [173]. On the other hand, it was the same in the serum of both UC patients and the control group [174]. Different interleukins control the migration of immune cells at the site of inflammation. For example, IL-8 acts as a chemoattractant, which induces the migration of neutrophils from peripheral blood to inflamed tissue [175]. Various studies have shown that IL-6 and its soluble receptors are increased in UC patients and play an essential role in the pathogenesis of UC and colorectal cancers related to UC [176]. T cells, part of adaptive immunity, also participate in UC [177]. T cells differentiate into Th17 cells when they encounter inflammatory mediators in the GI. Naive T cells also differentiate into regulatory T cells, Th1, Th2, and Th17, through a controlled process [178]. In a healthy state, the intestinal mucosa controls inflammation by Th1, Th17, Th2, Th3, Th9, and T reg cells. The breakage of self-antigen tolerance in the intestinal mucosa, by injury or genetic predisposition, may lead to UC [179]. In UC, there is a substantial increase in the secretion of IL-13, the main interleukin responsible for the inflammation and chronicity of this condition [180]. Despite Th1 involvement, UC patients also present a Th2 response, with increased secretion of IL-4, IL-5, and IL-9 [181,182]. The secretion of specific cytokines such as TNF-α [183,184,185], transforming growth factor-beta (TGF-β) [186,187], and interferon-gamma (IFN-γ) [188,189], as well as the response to self-antigens, are responsible for the development of UC [190,191]. 

Different clinical [192] and preclinical [193,194] studies recently identified that ECM degradation precedes inflammation in UC. These studies suggest that dysregulated ECM production and degradation leads to inflammation and pathogenesis in UC. A large body of evidence supports that the intestinal tissue of IBD patients overexpresses ECM remodeling enzymes, including matrix metalloproteases, heparanases, and elastases [195,196,197,198]. Secretion of these enzymes increases epithelial permeability and proinflammatory signaling loops and promotes colitis-associated tumorigenesis in mice [199,200].

## 5. Risk of Colorectal Cancer in UC

It is now well established that not only genetic factors but also various environmental factors can induce the risk of cancer [201,202,203]. In 1925, Crohn and Rosenberg proposed a link between inflammatory bowel disease (IBD) and colorectal cancer (CRC) for the first time, and studies have since confirmed this association [204]. Although IBD accounts for only 1%-2% of all CRC cases, 10%-15% of deaths in IBD cases are due to CRC [205]. While it is widely recognized that the risk of CRC is significantly higher in the UC cohort compared to the general population, the incidence rate may vary based on geographical location and ethnicity [206,207,208,209,210]. A meta-analysis of 116 articles found 1698 CRC cases out of 54,478 UC patients [206]. This study also reported that the incidence rate was higher in the US and UK than in Scandinavia. 

A study showed ten new CRC cases after a follow-up of 689 UC patients in Florence between 1978 and 1992 [211]. Similarly, another study reported 36 colon and 13 rectal cancers after following up with 2672 patients [212]. In comparison, a report noted only 13 CRC cases among 1160 patients with UC in Denmark [213]. As it is difficult to follow up with UC patients to account for the development of CRC, many studies have reported a distribution of incidence rates, which we summarized in Table 2.

The inflammation caused by UC is the primary factor that leads to the progression of the disease and is therefore considered a risk factor for the development of CRC [216]. While most cases of sporadic CRC arise from a preceding adenoma and are associated with unique genetic changes, an increased risk of CRC is believed to be an acquired event in inflammatory bowel disease (IBD) [217]. Studies suggest that various environmental factors, including diet and the microbiota, contribute to this risk [217]. Additionally, several studies have suggested various ways to reduce the risk of CRC development in UC patients. In clinical practice, colonoscopy surveillance is routinely recommended for early detection of CRC in UC patients, which can help in subsequent treatment [218]. However, it should be noted that colonoscopic surveillance does not reduce the risk of CRC.

## 6. Conclusions

**Figure 6:**
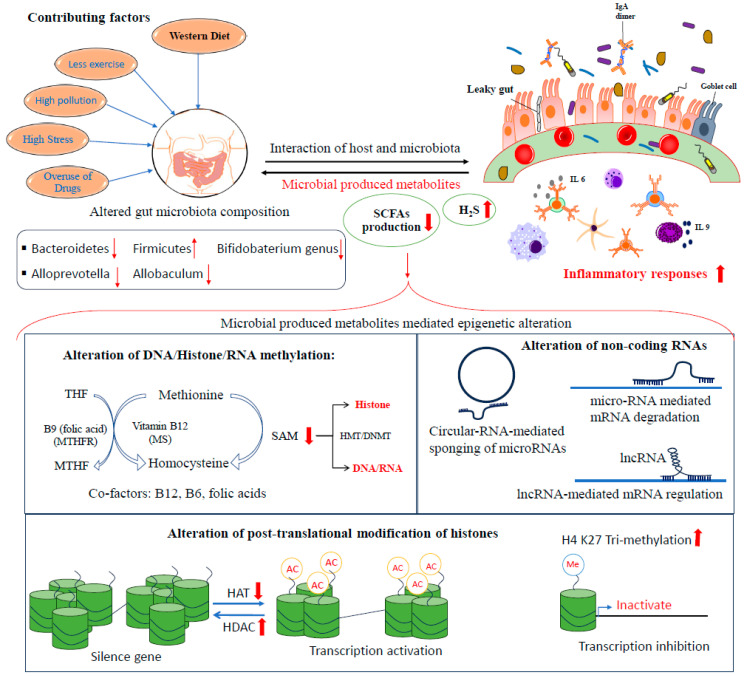
This figure schematically represents how the Western-type diet and other contributing factors cause colonic inflammation by changing the gut microbiota composition. This change can modulate the production of microbiota-produced metabolites, which then cause epigenetic changes in the host genome, thereby inducing inflammatory responses in UC.

A critical analysis of the available literature on UC shows that various factors play essential roles in UC development, but the factors connected with the health and growth of the gut microflora are most important, as shown in Figure 6. Studies have shown that the microflora is essential for the physical and physiological health of the gut, as well as the factors that kill beneficial bacteria and give harmful bacteria the chance to grow, resulting in the activation of immune cells. This consequently leads to UC and other complicated diseases, like colorectal cancer. 

## Figures and Tables

**Table 1:** Major types of cultivable bacteria found in the feces of an adult person [83].

Type of Bacteria	Genus	Approx. Count gm/Feces
Gram-negative bacilli	*Bacteroides*	10^10^
	*Fusobacterium*	10^9^
Gram-positive bacilli	*Eubacterium*	10^10^
	*Bifidobacterium*	10^10^
	*Lactobacillus*	10^4–8^
	*Clostridium*	10^10^
Cocci Gram-negative and Gram-positive	*Ruminococcus*	10^10^
	*peptostreptococcus*	
	*anaerobic streptococcus*	
Facultative anaerobic Enterobacteriaceae	*E. coli*	10^10^
	*Citrobacter*	
	*Enterobacter*	
	*Proteus*	
	*Klebsiella*	
	*Pathogen eg Shigella*, *Salmonella*, *Yersinia*	

**Table 2:** Follow-up of CRC cases in UC patients.

Number of UC Patients	Developed CRC Cases	Follow-Up Period (Person-Years)	Annual Crude Incidence (%)	Cumulative Incidence at 30 Years (%)	Study Location	Reference
2672	36	1984–1997 (19,655)	0.16	Not reported	Manitoba, Canada	[212]
378	6	1940–2004 (5567)	0.10	2	Olmsted County, USA	[214]
1160	13	1962–1987 (22,290)	0.06	2.1	Copenhagen County, Denmark	[213]
689	10	1978–1992 (7877)	0.12	Not reported	Florence, Italy	[211]
723	13	1974–2004 (8564)	0.15	7.5	Veszprem, Hungary	[215]
2672	13 rectum	1984–1997 (19,655)	0.06	Not reported	Manitoba, Canada	[212]
